# Supplementary material for: Social complexity, life-history and lineage influence the molecular basis of castes in vespid wasps
Source: Nat Commun. 2023 Feb 24;14:1046. doi: 10.1038/s41467-023-36456-6 (PMC9958023; doi:10.1038/s41467-023-36456-6)
Supplement: Supplementary file 1 — Supplementary Information [file 41467_2023_36456_MOESM1_ESM.pdf]

Supplementary Information

Supplementary Note 1: Hypergeometric test of pairwise differential expressed genes (DEGs)

To check for the significance of overlap in DEGs, we compared the orthologous genes (from Orthofinder) to measure significance of overlap of the pair-wise lists. This showed that for most pairwise comparisons we had significant overlap of the hypergeometric Bonferroni corrected P-values (Supplementary Information Figure 6). This suggests that the overlap is likely due to the same genes being important to for caste differentiation since the shared common ancestor of the two species. Noticeably, Agelaia does not have significant overlap in many of the pairwise comparisons.

Supplementary Note 2: Rates of gene evolution differ between colony founding strategies

We found that the influence of colony founding behaviour on molecular processes was different when comparing the rates of protein evolution in the swarm-founders relative to the independent founding species (Supplementary Data 7, tab ST12): the rates of protein evolution are on average higher among the swarm-founders (mean = 0.1461955) than the species with complex societies, but smaller than species with simple societies (Wilcoxon tests W = 384912, p-value = 1.847e-07, and W = 126972, p-value = 0.0001657 respectively). These results suggest that there may be additional caste-related molecular processes involved in the evolution of innovations in life-history traits (in this case, mode of colony founding) that are independent of social complexity.

Supplementary Figures

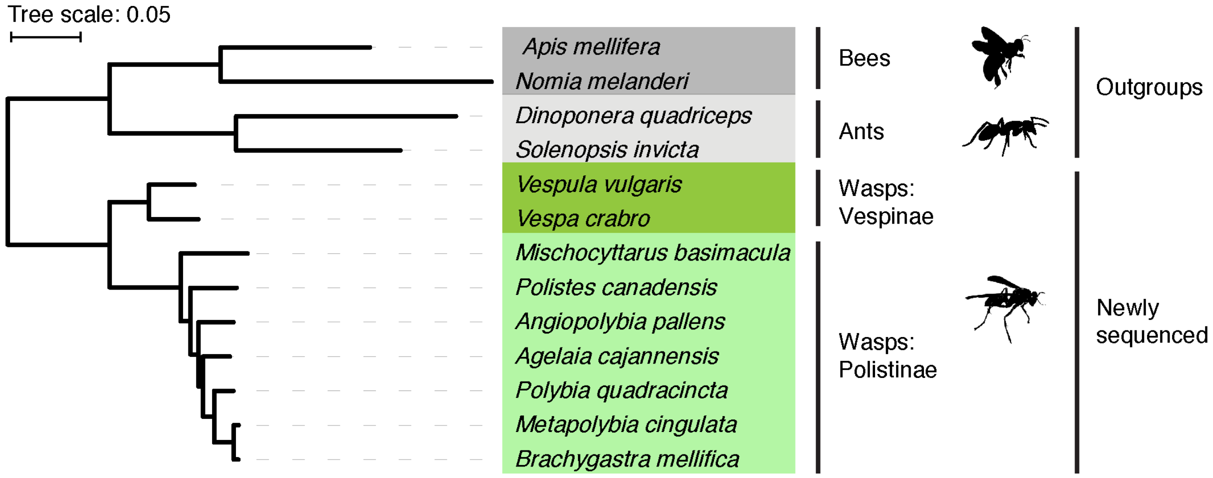

Supplementary Figure 1 | Phylogenetic tree of social wasps used in this study, and related hymenopterans generated using Orthofinder (SpeciesTree_rooted.txt). *Drosophila* was chosen as the root of the species tree (not shown), with two representative ants (*Dinoponera quadriceps* and *Solenopsis invicta*) and two bees (*Apis mellifera* and *Nomia melanderi*). Colours show groupings of ants, bees and wasps (Vespinae or Polistinae). For the nine wasp species (this study) we have

sequenced adult caste-specific brain transcriptomic data (queen and worker). As expected: Vespinae are clearly separated from the Polistinae; *Angiopolybia* is basal to the other Epiponini; independent-founding, non-superorganismal wasps (*Polistes* and *Mischocyttarus*) are basal to the Polistinae[1,2]. Insect silhouettes have creative commons licenses (wasp, ant) or no attribution pixabay licenses (bee).

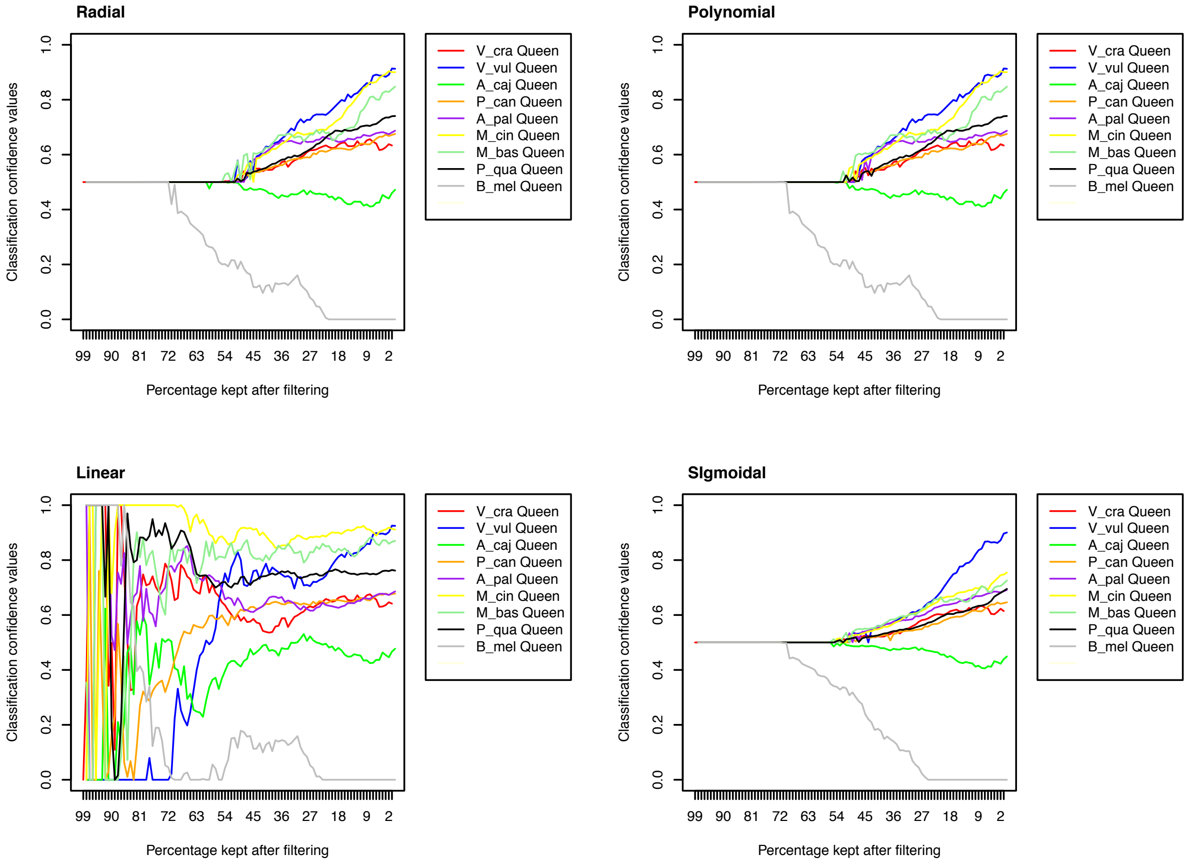

Supplementary Figure 2 | SVM kernel type iterations. For each of the four kernel types, a filtered SVM plot shows the leave one out experiment. Each coloured line represents the prediction of a queen sample(of the 9 species) at various regression filters, from 99% data inclusion to 1% inclusion (x-axis). Species are listed by a short code, representing their Genus (capital letter) and species (first three lower case letters of each species).

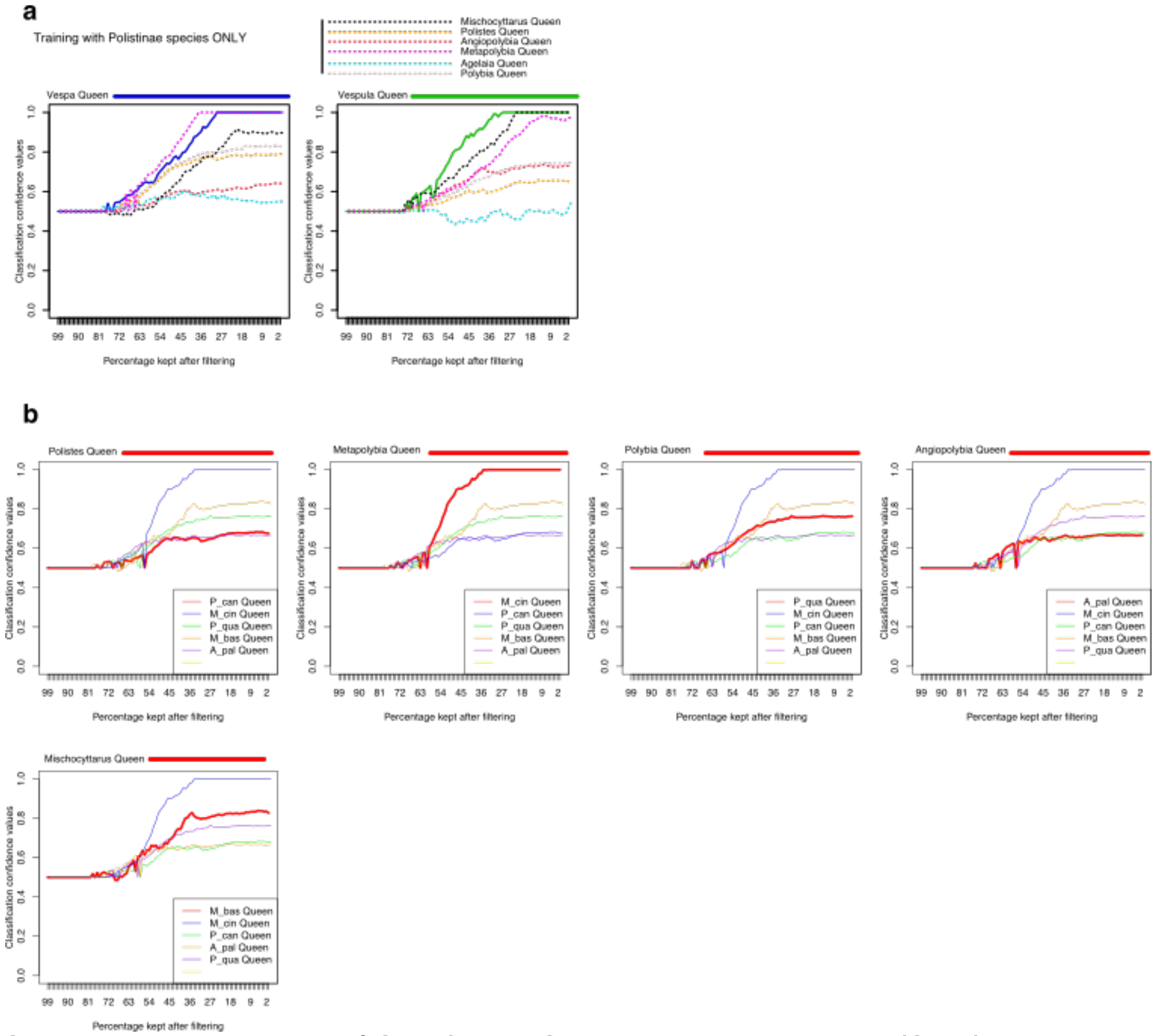

Supplementary Figure 3 | Sub-family Support vector machine (SVM) classifications. SVM classifications are given for each single species given training on species listed within the respective keys. Each coloured line represents the prediction of the queen sample at various regression filters, from 99% data inclusion to 1% inclusion of only the top genes in the regression analysis. **a)** Training with Polistine species only, and testing on the Vespines (*Vespa* and *Vespula*). The reverse was not possible, as the SVM requires a minimum number of samples to work**. b)** Training with 4 background polistine species and testing on the remaining Polistine wasp (always coloured red), with the training species in different colours (see key to determine species identity).

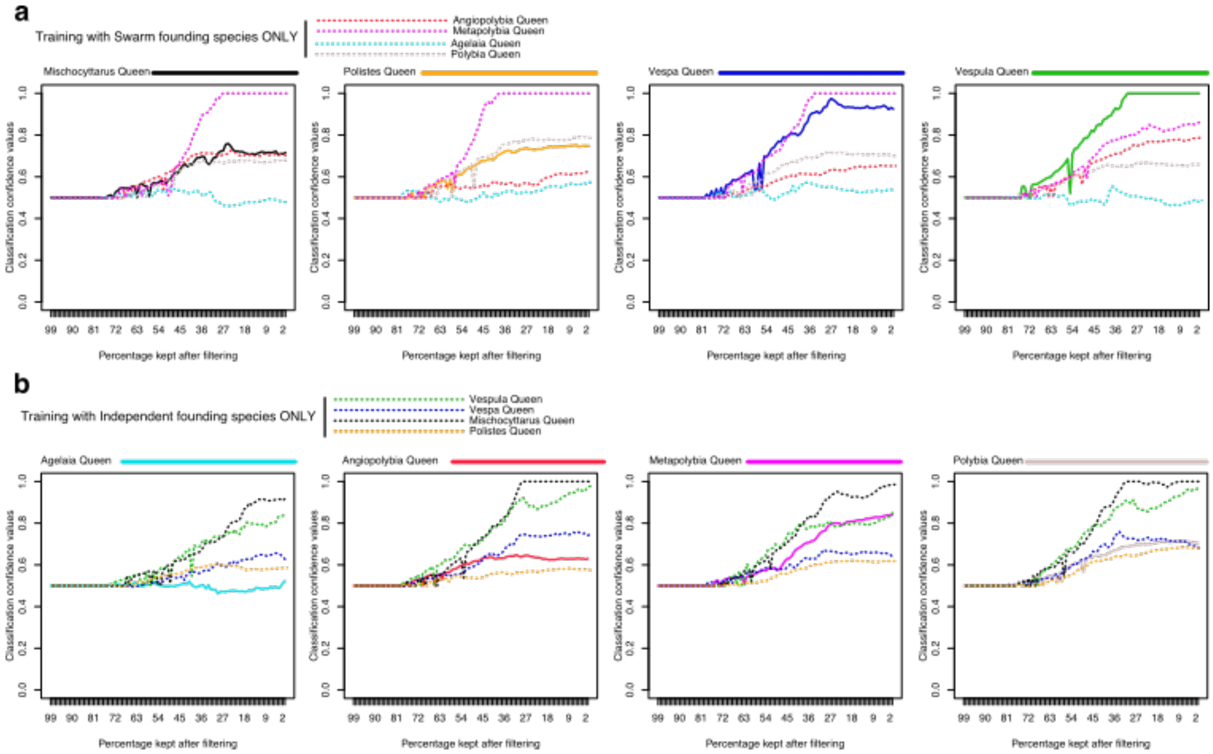

Supplementary Figure 4. | SVM classifications using founding behaviour and phylogeny to subsets of the training data. SVM classifications are given for each single species given training on species listed to left, showing the classification confidence after progressive feature selection from 95 to 1% of genes remaining after selection. Numbers of genes in each test are indicated in the lower left part of each plot**. a)** Training with independent-founders only, tested on the four swarm-founders (see Fig. 1 for species). **b)** Training with swarm-founders and testing on the four independent-founders.

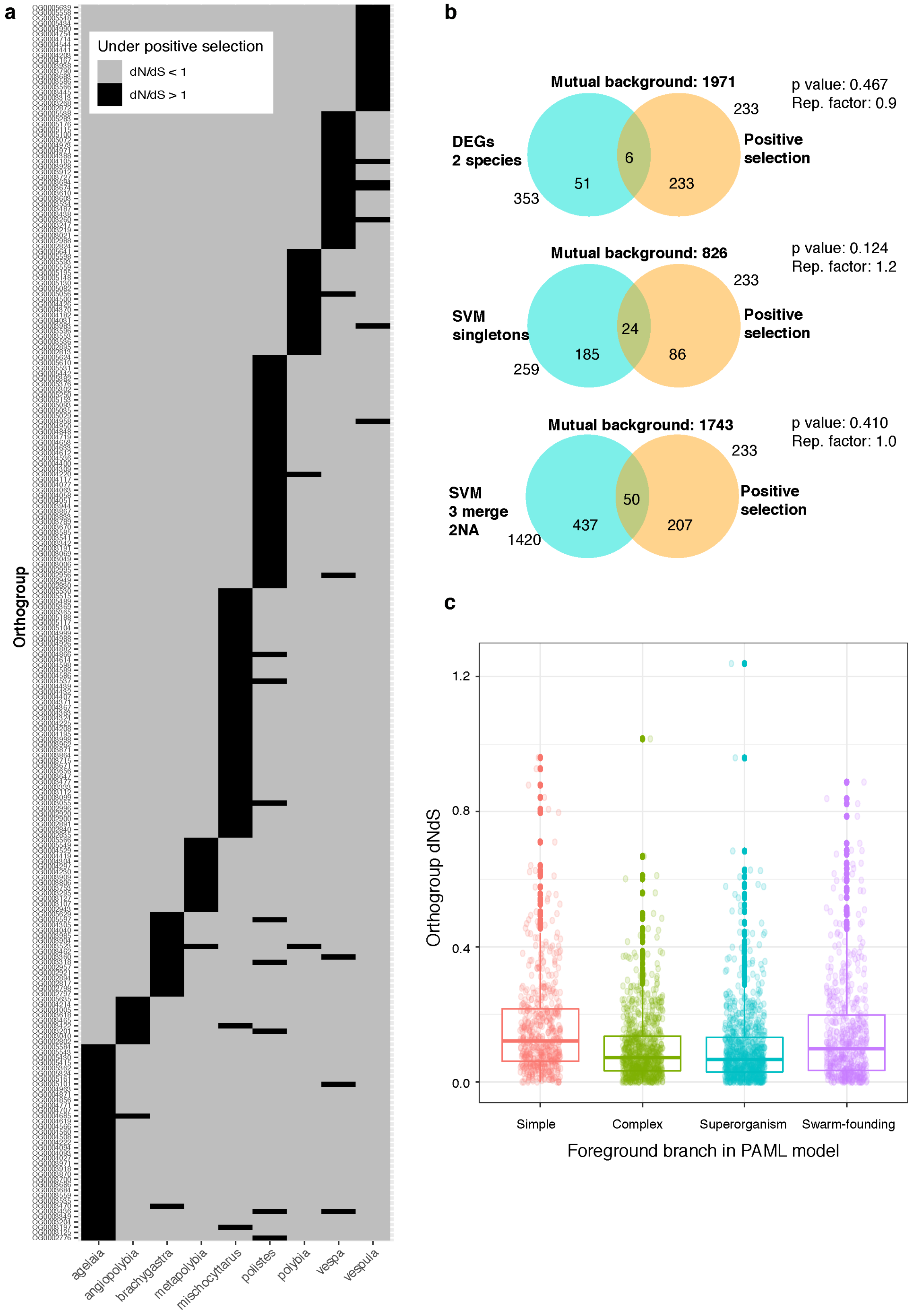

Supplementary Figure 5 | dNdS. **a)** All orthogroups that had at least one gene significantly under selection are listed (branch-site models). **b)** Overlap of positive selection genes and various toolkit gene lists. Top, shows the differential gene list overlap. Middle, shows the SVM gene list <0.05 for singleton orthogroups. Bottom, shows the three gene isoform merge and 2 NA gene list. The number of genes overlapping are in the middle and in each sphere shows the total number of genes

compared for each list (given that the two backgrounds do not overlap). A mutual background is listed showing the total number of genes that were possible to have been significant in the two compared lists. **c)** Comparison of four positive selection tests. Each box plot represents four models that were each run on the nine species, with different foreground branches highlighted, namely: Complex (*Vespa*, *Vespula*, *Brachygastra*, *Polybia*), Superorganism (*Vespa*, *Vespula*, *Brachygastra*), Swarm-founding (*Angiopolybia*, *Agelaia*, *Polybia*, *Metapolybia*, *Brachygastra*), Totipotent (*Mischocyttarus*, *Polistes*, *Angiopolybia*, *Metapolybia*). The box plot minima and maxima represent the smallest and largest values excluding outliers; the center represents the median. The lower and upper hinges of the box plot represents 25th and 75th percentiles; each whisker represents all values from the percentile to no further than 1.5 × interquartile range from the hinge. The sample size is 1,971 orthogroups shared between 9 species. Each dot represents the dN/dS ratio (ω) for a given orthogroup, for which the alternative model (PAML codeml's branch site model = 2, Nsites = 0) fitted the data best (i.e. ChiSquare Test *P*-value < 0.05). The mean of each category is: Superorganism = 0.1009398; Complex = 0.1015024; Swarm = 0.1461955; Totipotent = 0.1639218. Wilcoxon's tests show significant difference between swarm-founding and any other category (Complex/Superorganism W = 384912, *P*-value = 1.847e-07; Totipotent W = 126972, *P*-value = 0.0001657). There is also a significant difference between Totipotent and Complex (W = 307318, *P*-value < 2.2e-16). There is no significant difference between Complex and Superorganism (W = 356580, *P*-value = 0.1521).

	Agelaia_cajannensis	Angiopolybia_pallens	Brachygastra_mellifica	Metapolybia_cingulata	Mischocyttarus_basimacula	Polistes_canadensis	Polybia_quadracincta	Vespa_crabro	Vespula_vulgaris
Angiopolybia_pallens	0.0057913								
Brachygastra_mellifica	0.0006541	3.99E-13							
Metapolybia_cingulata	0.0001048	2.82E-17	1.27E-61						
Mischocyttarus_basimacula	8.14E-05	1.29E-22	1.47E-44	3.79E-41					
Polistes_canadensis	0.0013847	2.20E-07	1.06E-07	9.42E-15	8.58E-15				
Polybia_quadracincta	0.1588013	6.82E-05	1.37E-14	8.53E-19	2.21E-13	3.94E-14			
Vespa_crabro	3.07E-05	7.05E-05	7.52E-18	1.55E-12	1.56E-13	1.10E-05	0.0009445		
Vespula_vulgaris	0.0851082	1.47E-09	5.54E-26	4.51E-39	1.21E-40	2.73E-15	1.34E-12	2.37E-16	

Supplementary Figure 6. Hypergeometric test of significant overlap between differentially expressed genes between the nine species. Each square shows Bonferroni corrected P-values for Hypergeometric tests. Dark green highlights pairs with low P-values, scaled to white, where we have P-values near 1.

Supplementary References

1. Menezes, R. S. T., Lloyd, M. W. & Brady, S. G. Phylogenomics indicates Amazonia as the major source of Neotropical swarm-founding social wasp diversity: Phylogenomics of epiponine wasps. *Proc. R. Soc. B Biol. Sci.* (2020). doi:10.1098/rspb.2020.0480rspb20200480
2. Piekarski, P. K., Carpenter, J. M., Lemmon, A. R., Lemmon, E. M. & Sharanowski, B. J. Phylogenomic evidence overturns current conceptions of social evolution in wasps (vespidae). *Mol. Biol. Evol.* **35**, 2097–2109 (2018).
