# Supplementary material for: Social complexity, life-history and lineage influence the molecular basis of castes in vespid wasps
Source: Nat Commun. 2023 Feb 24;14:1046. doi: 10.1038/s41467-023-36456-6 (PMC9958023; doi:10.1038/s41467-023-36456-6)
Supplement: Supplementary file 3 — Description of Additional Supplementary Files [file 41467_2023_36456_MOESM3_ESM.pdf]

Supplementary Data Legends

Supplementary Data 1: RNA-seq samples used in this study. See ReadMe Tab for more detail. For each species, we show the wasp and nest IDs, caste ID, Collection site, GEO ascension numbers along with the merging of data required to form a single Queen and Worker replicate per species (and names given to these merged datasets). The second tab shows Trinity assembly statistics for the nine species (default output of Trinity). See https://github.com/trinityrnaseq/trinityrnaseq/wiki/Transcriptome-Contig-Nx-and-ExN50-stats. Showing numbers of genes and transcripts per assembly. Then conventional Nx length statistics, showing lengths of transcripts at N10/20/30/40/50 (% of assembled bases). This is performed again on the longest isoform of each gene. Finally we provide some details of quality control explained in the Readme Tab.

Supplementary Data 2: Orthogroups. Trinity gene IDs for each species are listed along with their grouping into orthologous gene groups (Orthogroups: OG [A]; tabs B:J). These are followed by counts of isoforms per species per OG (Tabs K:T). The a score for whether the gene is single copy across all the species (col U).Columns â€œV:AEâ€ show the data after merging of three isoforms into the single most expressed isoform. Followed by count for single copy (AF-AO). Then score for single copy (col AP). The trinity lists for three merged isoforms plus 2 NAs (col AQ-AZ), followed by counts for single isoform for each species (BA-BJ), and a count for single copy orthogroups (BK). The next four columns show the Metapolybia blast hit (Trinity name blasted), Description of hit sequence, evalue and mean similarity (cols BL-BO).

Supplementary Data 3: Differential expression. Each orthogroup is tested (edgeR) to determine whether the representative gene for each species is upregulated in the reproductive (UP), down-regulated (DW), not-changing significantly (NA) or without a representative gene (NA). The sum of UP, DW and total are listed. (Tab 1 [Table of Orthogroups]). This information is extracted from the individual species edgeR results, shown in the next 9 tabs: Differentially expressed genes (9 tabs names: 1 for each species). Showing edgeR results with Trinity gene names, using hard coded dispersion of 2. Columns show standard edgeR output, with log fold change values (logFC), log counts per million (logCPM), p value between queen/worker and false-discovery rate adjusted p value (fdr). Finally, we show the genes differential in at least 4 species (either direction; tab name="DEGs_4+ species") and its TopGO result (GO_RESULT 4+; standard tests for each signficant term) and genes differential in at least two species in the same direction (e.g. both queen upregulated, or both worker upregulated; tab name= "DEGs_2+ species").

Supplementary Data 4: Normalised training data (Used in the SVMs). Results from the SVM model using all nine species. Tab1 shows, A: Orthogroup, B-J: Trinity gene names for each of the nine species used in the SVM model. J:Orthogroup. L: Blast hit (Metapolybia sequence), M-AD: showing the normalised and species normalised counts, AE/AF: Linear regression p and q coefficients, and AG-AP shows default blast2go hits for Metapolybia sequences (listed in AG), showing Seq description (top blast hit), the sequence length (AI), the number of hits (limited to 5 per sequence), Blast evalue (AK), mean similarity in % (AL). Tab 2 shows the GO result (TopGO) , of the 400 significant genes across the nine species (p value <0.05).

Default output of TopGO. Bonferroni used in main figures.

Supplementary Data 5: SVM results for the subsection SVM models with each tab showing the SVM result for each species, with a background of four either simple or complex social species. colA: line number, colB: Orthogroup name, colC-L: showing the normalised counts, colM-T: showing species-normalised and scaled counts, colU-V: Linear regression p and q coefficients. The final two tabs show the Gene Ontology results for the simple and complex society "putative" toolkits, showing standard TopGO results files.

Supplementary Data 6: SVM results for the subsection SVM models with each tab showing the SVM result for each species, with a background of four either swarm-founding or independent-founding species (first 8 tabs). colA: line number, colB: Orthogroup name, colC-L: showing the normalised counts, colM-T: showing species-normalised and scaled counts, colU-V: Linear regression p and q coefficients. The next two tabs (Vespula_Polistinae/Vespa_Polistinae) show subsections using the Polistinae species as a training set , testing on either Vespa or Vespula. colA: line number, colB: Orthogroup name, colC-P: showing the normalised counts, colQ-AB: showing species-normalised and scaled counts, colAC-AD: Linear regression p and q coefficients. The final five tabs (vs Polistinae) show the experiments within the Polistines, with 1 species the test and the remaining four species the training set. Showing column headers the same as in the "Polistinae_section".

Supplementary. Table 7: DnDs: The first tab shows the summary of the DnDs experiment, followed by 25 PAML results for each of the comparisons and finally the last tab shows the information about the 11 Orthogroups that were significant in the three tests (The 6 in simple to complex polistines; the 4 among the more complex polistines and the 1 when we included the vespines). Below we describe the contents of each tab (Tab).

Tab ST1: Summary of dN/dS analyses with hypotheses, model type, input tree

Tab ST2: PAML results from branch model with Agelaia as foreground
Summary: This and each subsequent table has the following column headings. Name of gene (orthogroup), dNdS ratio in null model (w_M0), number of parameters in null model (np_M0), log likelihood in null model (lnL_M0), number of parameters in alternative model (np_M2), log likelihood in alternative modell (nL_M2), dNdS ratio on background branch (background_w), dNdS ratio on foreground branch (foreground_w), transition/transversion ratio (kappa_M2), test statistic (D), degree of freedom (DF), likelihood ratio test p-value (chiTest)

Tab ST3: PAML results from branch model with Angiopolybia as foreground

Tab ST4: PAML results from branch model with Brachygastra as foreground

Tab ST5: PAML results from branch model with Metapolybia as foreground

Tab ST6: PAML results from branch model with Mischocyttarus as foreground

Tab ST7: PAML results from branch model with Polistes as foreground

Tab ST8: PAML results from branch model with Polybia as foreground

Tab ST9: PAML results from branch model with Vespa as foreground

Tab ST10: PAML results from branch model with Vespula as foreground

Tab ST11: PAML results from branch model with Vespines as foreground

Tab ST12: PAML results from branch model with Swarm-founding species as foreground

Tab ST13: PAML results from branch model with Simple species as foreground

Tab ST14: PAML results from branch model with Complex species as foreground

Tab ST15: PAML results from branch model with Simple species as foreground (Polistines only; Vespines excluded)

Tab ST16: PAML results from branch model with Complex species as foreground (Polistines only; Vespines excluded)

Tab ST17: PAML results from branch-site model with Agelaia as foreground

Tab ST18: PAML results from branch-site model with Angiopolybia as foreground

Tab ST19: PAML results from branch-site model with Brachygastra as foreground

Tab ST20: PAML results from branch-site model with Metapolybia as foreground

Tab ST21: PAML results from branch-site model with Mischocyttarus as foreground

Tab ST22: PAML results from branch-site model with Polistes as foreground

Tab ST23: PAML results from branch-site model with Polybia as foreground

Tab ST24: PAML results from branch-site model with Vespa as foreground

Tab ST25: PAML results from branch-site model with Vespula as foreground

Tab ST26: PAML results from branch-site model with Vespa+Vespula as foreground

Tab ST27: Showing the 11 significant dnds orthogroups, with B:J showing whether the gene was differential in the DEG anlysis (UP,DW [down], or NA [not signficant or not tested]).ColK: Number of NAs. ColL-O, sum of all DEGs per species. ColP-X shows the Trinity ID for each species. ColY: dNdS branch information. ColZ: Google scholar hit. colAA-AB: Shows the pvalue for each orthogroup after linear regression in the analysis using 2 NAs and 3 isoform merges. ColAC-AG: show typical blast results for the Metapolybia gene representative of each orthogroup.

Supplementary Data 8: Morphometrics Data. The body parts measured were:

head length (HL), head width (HW), minimum interorbital distance (MID), partial forewing length (PWL), mesoscutum length (MSL), mesoscutum width (MSW), mesosoma height (MSH), alitrunk length (AL), length of tergite I (T1L), basal width of tergite II (T2BW), apical width of tergite II (T2AW), length of tergite II (T2L), and apical height of tergite II (T2AH). In Metapolybia, we also measured pronotal width, gena width, eye width, mesoscutellar length and mana interorbital distance. For each species we then conducted ANOVA, on each colony separately and for the listed characters.

Supplementary Data 9: Each cell contains the 8 crossfold validation error of all samples minus the listed Species in the header.
We conducted the error test across the learning curve using the feature selection filter, going from 99% of the data to 1 % of the data kept after feature selection.
We also show an example plot, plotting the cross fold error score, at each feature selection percentage from 99-1% retained.
