# Supplementary material for: Social complexity, life-history and lineage influence the molecular basis of castes in vespid wasps
Source: Nat Commun. 2023 Feb 24;14:1046. doi: 10.1038/s41467-023-36456-6 (PMC9958023; doi:10.1038/s41467-023-36456-6)
Supplement: Supplementary file 13 — Reporting Summary [file 41467_2023_36456_MOESM13_ESM.pdf]

nature portfolio

Corresponding author(s): *Double-blind peer review submissions: write DBPR and your manuscript number here instead of author names.*

Last updated by author(s): 2023/01/12

Reporting Summary

Nature Portfolio wishes to improve the reproducibility of the work that we publish. This form provides structure for consistency and transparency in reporting. For further information on Nature Portfolio policies, see our Editorial Policies and the Editorial Policy Checklist.

Statistics

For all statistical analyses, confirm that the following items are present in the figure legend, table legend, main text, or Methods section.

n/a	Confirmed	
☐	☒	The exact sample size (*n*) for each experimental group/condition, given as a discrete number and unit of measurement
☐	☒	A statement on whether measurements were taken from distinct samples or whether the same sample was measured repeatedly
☐	☒	The statistical test(s) used AND whether they are one- or two-sided<br>*Only common tests should be described solely by name; describe more complex techniques in the Methods section.*
☐	☒	A description of all covariates tested
☐	☒	A description of any assumptions or corrections, such as tests of normality and adjustment for multiple comparisons
☐	☒	A full description of the statistical parameters including central tendency (e.g. means) or other basic estimates (e.g. regression coefficient) AND variation (e.g. standard deviation) or associated estimates of uncertainty (e.g. confidence intervals)
☐	☒	For null hypothesis testing, the test statistic (e.g. *F*, *t*, *r*) with confidence intervals, effect sizes, degrees of freedom and *P* value noted<br>*Give P values as exact values whenever suitable.*
☐	☒	For Bayesian analysis, information on the choice of priors and Markov chain Monte Carlo settings
☐	☒	For hierarchical and complex designs, identification of the appropriate level for tests and full reporting of outcomes
☐	☒	Estimates of effect sizes (e.g. Cohen's *d*, Pearson's *r*), indicating how they were calculated

Our web collection on statistics for biologists contains articles on many of the points above.

Software and code

Policy information about availability of computer code

Data collection	Not applicable
Data analysis	GXCAM-1.3<br>GXCapture V8.0<br>SPSS v23.0<br>Exlstat 2018<br>BBTools (version:BBMap_38)<br>Trimmomatic v0.39<br>Trinity v2.8 and v2.8.4<br>Skewer v0.2.2<br>TransDecoder v5.5.0<br>Orthofinder v.2.2.7<br>diamond blast v2.0.13<br>Muscle v3.8.31<br>FastTree v2.1.10<br>RSEM v1.3.1<br>bowtie2 (default call within Trinity)<br>edgeR v3.26.5 ; R version 3.6.0<br>blast v2.11.0<br>nextflow version 21.10.4<br>PRANK (version v.15112089)

PAML v.4.8
topGO v2.42.0
Ensembl Biomart (date:1.10.2019)
Blast2GO v1.4.4

R packages (default version within Rv3.6.0)
e1071 v1.7-12
probsvm v1.00
edgeR v3.16
seqinr v4.2-23
stringr v1.5.0
pheatmap v1.0.12
tximport v3.16
tximportData c3.16
R v3.6.3 for DnDs analysis
R v3.6.0 for SVM analysis

For manuscripts utilizing custom algorithms or software that are central to the research but not yet described in published literature, software must be made available to editors and reviewers. We strongly encourage code deposition in a community repository (e.g. GitHub). See the Nature Portfolio guidelines for submitting code & software for further information.

Data

Policy information about availability of data

All manuscripts must include a data availability statement. This statement should provide the following information, where applicable:

- Accession codes, unique identifiers, or web links for publicly available datasets
- A description of any restrictions on data availability
- For clinical datasets or third party data, please ensure that the statement adheres to our policy

The raw sequencing (RNA-Seq) data generated in this study have been deposited in the NCBI GEO (Gene Expression Omnibus) database under accession code GSE159973 [https://www.ncbi.nlm.nih.gov/geo/query/acc.cgi?acc=GSE159973]. The processed orthology are provided in the Supplementary Data files and normalised Trinity count data are found within GSE159973 [https://www.ncbi.nlm.nih.gov/geo/query/acc.cgi?acc=GSE159973].

Human research participants

Policy information about studies involving human research participants and Sex and Gender in Research.

Reporting on sex and gender	*Use the terms sex (biological attribute) and gender (shaped by social and cultural circumstances) carefully in order to avoid confusing both terms. Indicate if findings apply to only one sex or gender; describe whether sex and gender were considered in study design whether sex and/or gender was determined based on self-reporting or assigned and methods used. Provide in the source data disaggregated sex and gender data where this information has been collected, and consent has been obtained for sharing of individual-level data; provide overall numbers in this Reporting Summary. Please state if this information has not been collected. Report sex- and gender-based analyses where performed, justify reasons for lack of sex- and gender-based analysis.*
Population characteristics	*Describe the covariate-relevant population characteristics of the human research participants (e.g. age, genotypic information, past and current diagnosis and treatment categories). If you filled out the behavioural & social sciences study design questions and have nothing to add here, write "See above."*
Recruitment	*Describe how participants were recruited. Outline any potential self-selection bias or other biases that may be present and how these are likely to impact results.*
Ethics oversight	*Identify the organization(s) that approved the study protocol.*

Note that full information on the approval of the study protocol must also be provided in the manuscript.

Field-specific reporting

Please select the one below that is the best fit for your research. If you are not sure, read the appropriate sections before making your selection.

☒ Life sciences ☐ Behavioural & social sciences ☐ Ecological, evolutionary & environmental sciences

For a reference copy of the document with all sections, see nature.com/documents/nr-reporting-summary-flat.pdf

Life sciences study design

All studies must disclose on these points even when the disclosure is negative.

Sample size	The sample size for each species caste was 1. This was due to availability of wasp brain samples, however we merged multiple individuals into a single replicate per caste per species.
Data exclusions	No data were excluded, except for the removal Brachygastra from some SVM training sets for our predictions, to ensure test parity in terms of the number of replicates in the training set
Replication	Findings were not replicated due to difficulty collecting the samples. We did however merge multiple invidividuals to reduce the effect of individual level variation for each species, and also to collect enough RNA for sucesssful processing.
Randomization	Not relevant
Blinding	Blinding was not conducted. We needed to ensure each sample was labeled correctly, with its species and caste identity.

Reporting for specific materials, systems and methods

We require information from authors about some types of materials, experimental systems and methods used in many studies. Here, indicate whether each material, system or method listed is relevant to your study. If you are not sure if a list item applies to your research, read the appropriate section before selecting a response.

Materials & experimental systems

n/a	Involved in the study	
☒	☐	Antibodies
☒	☐	Eukaryotic cell lines
☒	☐	Palaeontology and archaeology
☐	☒	Animals and other organisms
☒	☐	Clinical data
☒	☐	Dual use research of concern

Methods

n/a	Involved in the study	
☒	☐	ChIP-seq
☒	☐	Flow cytometry
☒	☐	MRI-based neuroimaging

Animals and other research organisms

Policy information about studies involving animals; ARRIVE guidelines recommended for reporting animal research, and Sex and Gender in Research

Laboratory animals	Study did not involve lab animals
Wild animals	All colonies used in this study were post-emergence, meaning they were well established, with brood of all life stages (eggs, larvae and pupae) and were actively producing adults. Where possible, we sampled from colonies representing different stages in the colony cycle, as caste differentiation can vary as the colony matures in some species. All species were collected in the same way, as whole colonies collected in situ in the wild at a time of day when foraging was actively taking place.
Reporting on sex	Study involves only female individuals, as we were specifically comparing two female castes.
Field-collected samples	All colonies were collected in a similar way, with wasps picked from the nest and placed immediately onto dry ice or directly into RNAlater (Ambion, Invitrogen) and stored at -20°C until further use.
Ethics oversight	No ethical guidance was required

Note that full information on the approval of the study protocol must also be provided in the manuscript.
